# *Honduranura centraliamericana* gen. n. et sp. n. from Central America (Collembola, Neanuridae, Neanurinae)

**DOI:** 10.3897/zookeys.723.12258

**Published:** 2017-12-18

**Authors:** José G. Palacios-Vargas

**Affiliations:** 1 Laboratorio de Ecología y Sistemática de Microartrópodos, Departamento de Ecología y Recursos Naturales, Facultad de Ciencias, Universidad Nacional Autónoma de México, 04510 México, D.F., México

**Keywords:** Honduras, Costa Rica, morphology, Sensillanurini, taxonomy

## Abstract

*Honduranura* **gen. n.** and the type species *H. centraliamericana* **sp. n.** are described and illustrated. The new genus shows the characters of Sensillanurini Cassagnau, 1983 tribe and is distinguished by the fusion of cephalic and abdominal tubercles: clypeal with antennofrontal and dorsointernal with dorsoexternal on head; presence of fused tubercles on each side of abdominal segment V. Most of the tubercles bear strong subcuticular reticulation. A key for the genera of the tribe Sensillanurini is provided.

## Introduction

To date, the tribe Sensillanurini contains only four genera: *Americanura* Cassagnau, 1983, with a wide distribution in North America, México, Central America, and the north of South America; *Palmanura* Cassagnau, 1983, with a Neotropical distribution from Central México to north of South America; *Sensillanura* Deharveng, 1981, with Holarctic and Nearctic distribution and *Tabasconura*, Palacios-Vargas & Catalán, 2015, endemic of Mexico. The tribe Sensillanurini has a high diversity of species in the Neotropical Region (46 out of 49 named species), and is characterized by hypertrophy of the sensillum S7 on antennal segment IV (Deharveng 1981; Palacios-Vargas et al. 2009), development of cuticular tubercles and reduction of the chaetotaxy. The new genus seems to be wide distributed in Central America (from Honduras to Costa Rica). It is distinguished by the fusion of some tubercles and the elongation of the dorsolateral and lateral ones on tergites.

## Materials and methods

Samples of leaf litter were collected at Camayagua, Honduras and processed by Berlese-Tullgren funnels. Specimens of Neanuridae were kept in 75 % alcohol and sent to the author. Members of the new genus were cleared and mounted in Hoyer’s solution under slides. Observations and measurements were made using a Carl Zeiss Axiostar Plus phase contrast microscope with an adapted drawing tube. Dorsal chaetotaxy follows Deharveng and Weiner (1984) modified by Palacios-Vargas and Catalán (2010) and ventral chaetotaxy follows Smolis (2008) and Smolis and Deharveng (2006).

### Abbreviations

**Abd** abdominal segment

**Af** cephalic antenno-frontal tubercle

**asl** above sea level

**Ant** antennal segment

**Cl** clypeal tubercle

**Cx** coxa

**Di** dorso-internal tubercle

**De** dorso-external tubercle

**DL** dorso-lateral tubercle

**Fe** femur

**Fu** furcal vestige

**L** lateral tubercle

**L**’ ordinary lateral seta on Abd. V

**M** macrosetae

**me** mesosetae

**mi** microseta

**m**’ ventral microsensillum of Ant III

**Oc** ocular tubercle

**Ocm** ocular median seta

**Ocp** ocular posterior seta

**or** subapical organ of Ant. IV

**S** cylindrical sensillum on Ant IV

**Scx2** subcoxa 2

**sgd** dorsal guard sensillum

**sgv** ventral guard sensillum

**So** sub-ocular tubercle

**ss** sensorial setae on body

**T** tibiotarsus

**Th.** thoracic segment

**Tr** trochanter

**VT** ventral tube

**Ve** ventroexternal

**Vel** ventroexternolateral

**Vec** ventroexternocentral

**Vei** ventroexternointernal

**Vi** ventrointernal

**Vl** ventrolateral.

## Results

### Taxonomy

#### Honduranura gen. n.

Taxon classificationAnimaliaCollembolaNeanuridae

http://zoobank.org/FC1B78AC-2830-40D6-861B-A532ED097BF2

##### Type species.

*Honduranura centraliamericana* sp. n.

##### Diagnosis.

**Figures 1–3.**
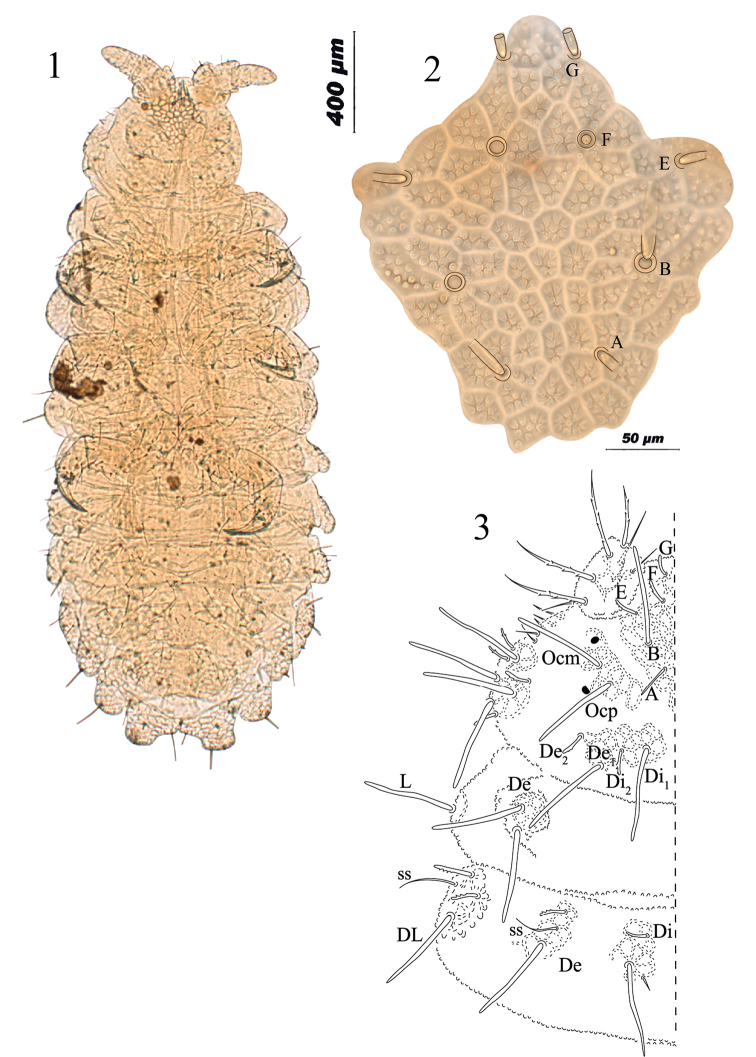
*Honduranura centraliamericana* sp. n. **1** habitus on slide **2** antennofrontal + clypeal tubercle **3** dorsal chaetotaxy of head and thoracic segments I–II.

Neanuridae with aspect of a yellow *Neanura*. 2+2 slightly pigmented big eyes. Body color yellow or orange when alive, without blue pigment, almost white in alcohol (Fig. 1). Mouthparts reduced, maxillae styliform. Sensilla S7 on Ant. IV hypertrophied, at least twice thicker than others. Clypeal and Antennofrontal tubercles fused altogether, cephalic setae A, B, E, F and G present (O, C and D absent) (Fig. 2). Two ocular setae: Ocm and Ocp. Posterior cephalic tubercles Di and De fused at each side, Di1, Di2, De1 and De2, not in crossed pattern. Dorsolateral tubercle (DL) separate, with two me and two M setae. Lateral tubercle with two M and two me, subocular tubercle with 5 setae. Thorax I with two M setae on De and one M seta on DL tubercle, without setae or tubercle on Di position. Di tubercle of Th. II and III with three setae, one M and two mi; Di tubercle on Abd. I–III with two setae, one M and one me. De tubercle with two setae one M and one me, plus ss from Th. II to Abd. III. Tubercle Di on Abd. IV with two setae M and mi, other tubercles with lateroexternal migration. Abd. V with tubercles Di, De and DL fused, with three setae and one sensorial seta ss. Four (2+2) macrosetae between the sensorial setae on Abd. IV, two (1+1) setae between sensorial setae of Abd. V. Head and body tubercles with strong subcuticular reticulation. Distal part of abdomen strongly bilobed.

##### Remarks.

This is the only genus in the Sensillanurini tribe with clypeal and antennofrontal tubercles fused on head, dorso-internal and dorso-external tubercles fused on each side of the head, and tubercles of abdomen V fused on each side of the body. In addition, all tubercles exhibit a strong subcuticular reticulation, and dorsolateral and lateral tubercles slightly elongated.

##### Etymology.

The name of genus is after the country where the author had seen specimens for the first time and it is the type locality of the type species. Gender of genus is feminine.

### Key for the genera of Sensillanurini

**Table:** 

1	Presence of Di tubercle and one seta on Th. I	***Sensillanura***
–	Lacking Di tubercle and seta on Th. I	**2**
2	Cephalic tubercles Di and De; Cl and Ant fused	***Honduranura* gen. n.**
–	Cephalic tubercles clearly isolate	**3**
3	Dorsal tubercles developed and “finger-like”; S2 hypertrophied and thickened similar to S7 on Ant. IV	***Tabasconura***
–	Dorsal tubercles not elongated; S2 not hypertrophied, thin and short, similar to others except S7	**4**
4	Cephalic tubercle De with 3‒1 setae, two or one setae on tubercle Di of Abd. IV and V; most dorsal macrosetae smooth or barbulate	***Americanura***
–	Cephalic tubercle De always with 1 seta; only one seta on tubercle Di of Abd. IV and V; most dorsal macrosetae palmate with serrate margins	***Palmanura***

#### Honduranura centraliamericana sp. n.

Taxon classificationAnimaliaCollembolaNeanuridae

http://zoobank.org/DF4C2585-74C5-4A18-AC62-B65C24B1E6F0

Figs 1–3 , 4–7 , 8–12 , Tables 1 , 2

##### Type material.

Holotype: adult female; Paratypes: three adult females, one adult male and one juvenile. All the type material kept at author’s institution.

##### Type locality.

Central America: Honduras: Camayagua (14°48'39"N; 87°53'22"W). 2140 m asl. FS2A LLAMA # Wa-C03-2-all, cloud forest, samples of leaf litter. 05.v.2010, F. Soto-Adames leg.

##### Other material.

Central America: Costa Rica: Sierra de Talamanca. Parque Nacional Tapantí (9°46'14"N; 83°47'59"W). 1200 m asl, tropical rain forest, *ex* rotting trunk. 19.vii.2010, J. G. Palacios-Vargas col. One female and one juvenile.

##### Description.

**Figures 4–7.**
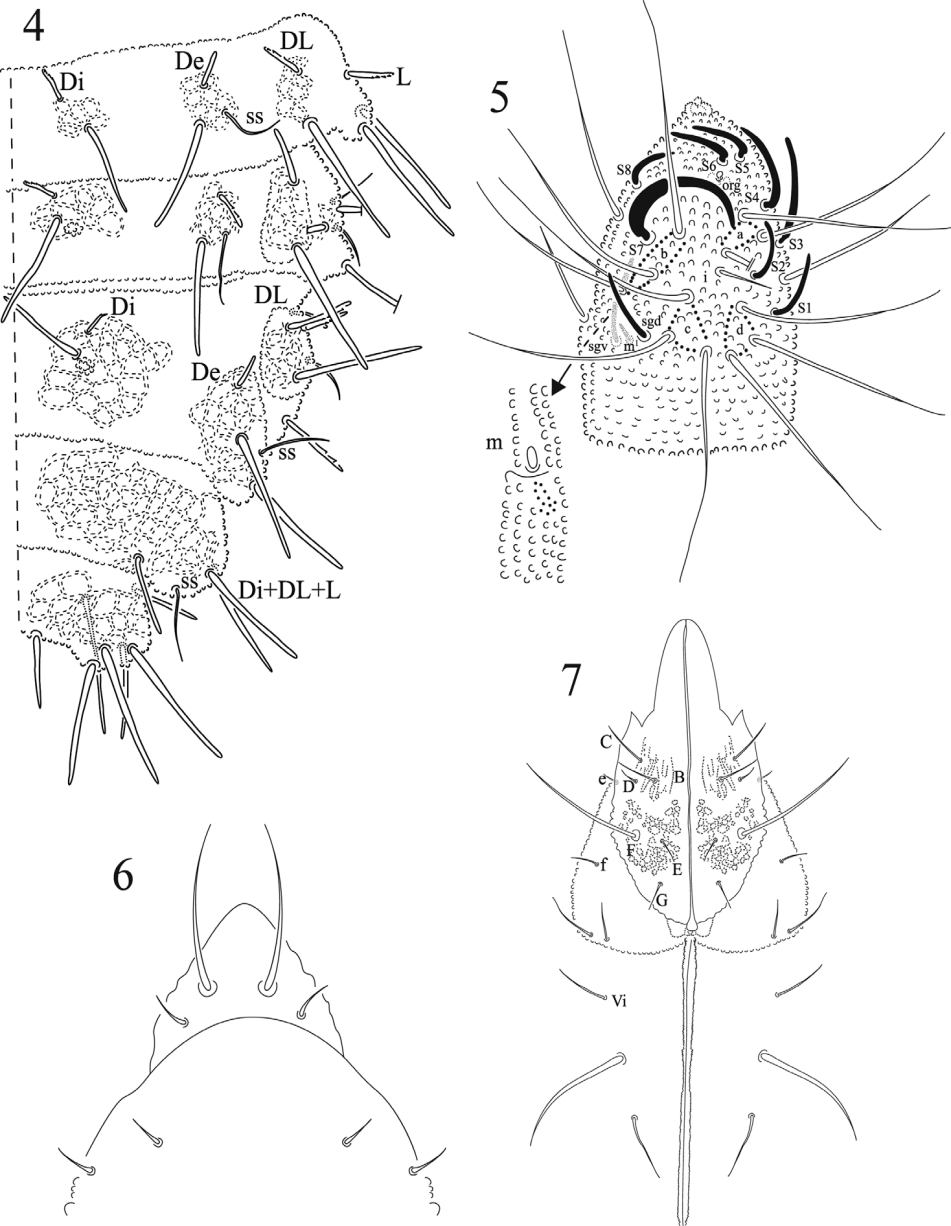
*Honduranura centraliamericana* sp. n. **4** dorsal chaetotaxy of abdominals segments II–VI **5** dorsal antennal segments III and IV **6** chaetotaxy of pre-labrum/labrum **7** chaetotaxy of labium.

Length of holotype 2.5 mm; length range: 2.2–2.8 mm (n = 5). Color yellowish. Granulations strong, approximately 1/4 diameter of one eye. Tubercles well developed mainly on lateral and posterior part of body (Fig. 1), with strong subcuticular reticulation. Head with clypeal and antennofrontal tubercles fused with setae A, B, E, F, G present (O, C and D absent) (Fig. 2); posterior cephalic tubercles dorsointernal and dorsoexternal fused (Fig. 3). On Abd. V there is only one tubercle on each side (Fig. 4). Two kinds of dorsal body setae, macrosetae (M) 46 µm (38–60 µm) with blunt apex, mesosetae (me) with blunt apex, both slightly serrate in both sides, besides sensorial setae (ss) (30 µm). All ventral setae are smooth and acuminate; some are macrosetae and most are mesosetae.

Ant. I with 9 setae, 4 dorsal slightly barbulate macrosetae on a surface with subcuticular reticulation. Ant. II with 11 setae, one of them slightly serrate. Ant. III sensorial organ with two globular sensilla in a cuticular fold, and two guard sensilla; S.g.d slightly curved, one microsensillum ventro-external. Ant. IV with hypertrofied sensilla S7; S2 like other sensilla. One clear subapical organite. Apical bulb of Ant. IV trilobed (Fig. 5).

Labrum with 4 short prelabral setae, two short basal setae and two long apical setae (Fig. 6). Labium without tuberculate seta L, organite “x” or seta A (as cited by Deharveng, 1983 for the subfamily) Seta D short; seta F much longer than E and G (Fig. 7). Eyes 2+2, large, with dark pigment. Mandibles with three teeth. Maxillae styliform. Head with clypeal and antennofrontal tubercles fused, ocular seta Ocm and Ocp in one independent tubercle, Di and De tubercles fused, DL and L tubercles independent and well developed. Head chaetotaxy in figure 3 and in Table 1. Three pairs of postlabial setae, the second one much larger and thicker than others (Fig. 7).

**Figures 8–12.**
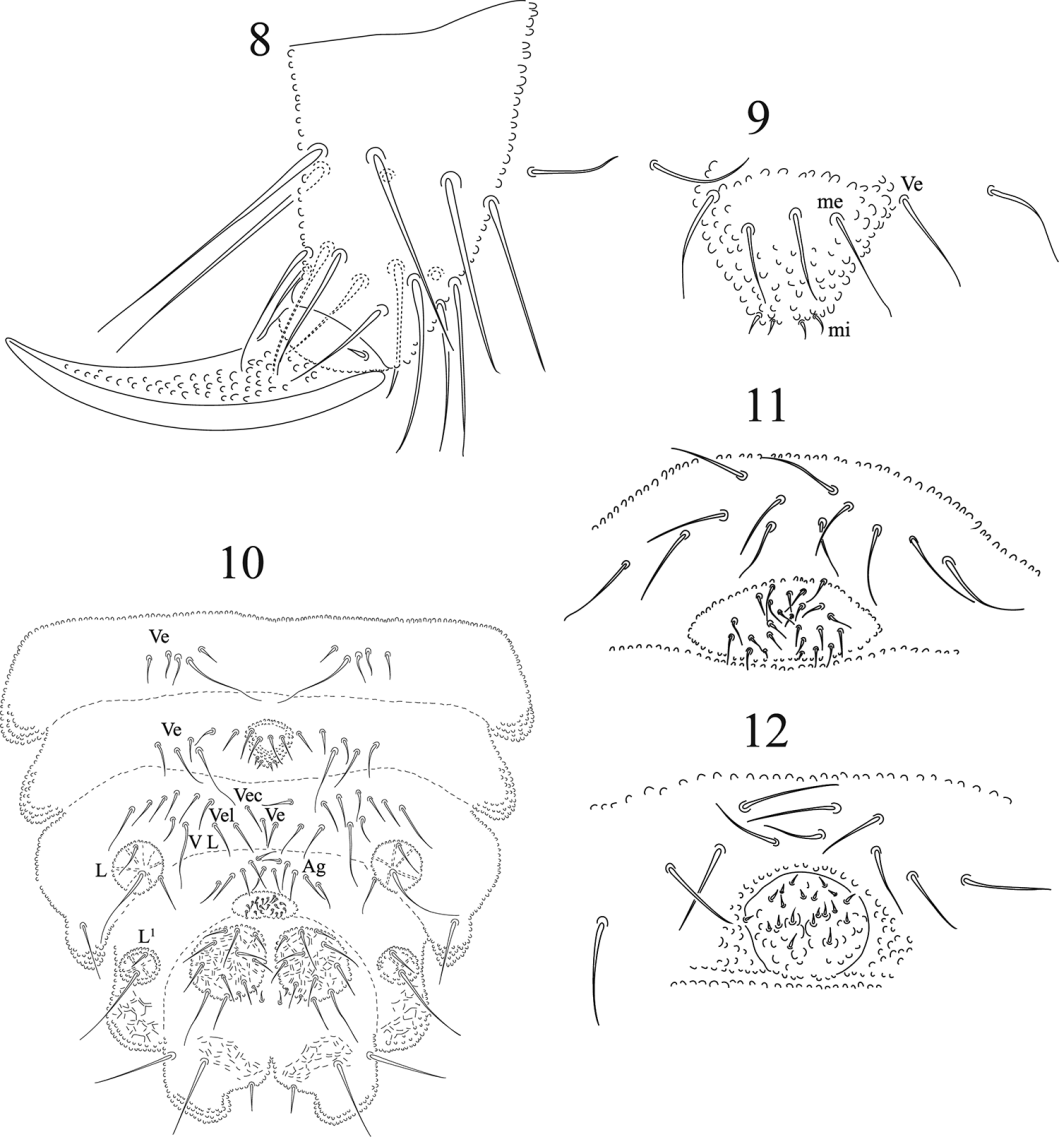
*Honduranura centraliamericana* sp. n. **8** lateral view of tibiotarsus III chaetotaxy **9** furcular vestige (midventral region of Abd. III) **10** ventral chaetotaxy of Abd. II–VI **11** female genital plate **12** male genital plate.

Legs chaetotaxy from coxae to tibiotarsi (I, II and III), respectively, as 3,7,7; 5,5,5; 10,10,10; 18, 18, and 17 setae, without capitate tenent hairs, but with setae B4 and B5 long and acuminate (Fig. 8). One ventral seta of trochanter is small and very thin. Each femur with one long ventral seta. Ungues with strong granulation but without tooth. Thoracic and abdominal chaetotaxy in Figs 3 and 4. Body chaetotaxy by half tergite is shown in Table 2.

**Table 1.** Head chaetotaxy of *Honduranura centraliamericana* sp. n.

Head setae group	Tubercles	Number of setae	Kind of setae	Setae
**Cl+Af**	1	5	1M, 4me	A, B, E, F, G
**Oc**	1	2	2M	Ocm, Ocp
**Di + De**	1	4	2M, 2me	Di1, Di2, De1, De2
**DL**	1	4	3M, 1 me	
**L**	1	4	1M, 3 me	
**So**	–	5	Mi	
**Total number**	5	24		

**Table 2.** Thorax and abdomen chaetotaxy of *Honduranura centraliamericana* sp. n. by half tergite.

Thorax & Abdomen Dorsal					Legs				
	Di	De	DL	L	Scx2	Cx	Tr	Fe	T
**Th. I**	–	2M	M		0	3	6	13	19
**Th. II**	M, 2m	M, me+ss	M, 2me +ss	M	2	7	6	12	19
**Th. III**	M, 2m	M, me+ss	M, 2me +ss	M	2	8	6	11	18
					**Abdomen Ventral**				
**Abd. I**	M, me	M, me+ss	1M, 1me	2M, 2me	VT: 4				
**Abd. II**	M, me	M, me+ss	1M, 1me	2M, 2me	Ve: 6(5)	Vel 0			
**Abd. III**	M, me	M, me+ss	1M, 1me	2M, 2me	Vel: 6(7)	–	–	Fu: 4me	4mi
**Abd. IV**	M, me	(2M,	me +ss)	M, 5me	Vel: 9	Vec: 2	Vei: 1	VI: 3	
**Abd. V**	(M +	ss, 2M)	M, me	M, me	Ag: 6	–	–	VI: 2	L: 1
**Abd. VI**	7–				Ve:12	–	–	An: 2mi	

Ventral tube with 4 + 4 setae, the two distal setae subequal in size, basal setae are different, one is larger. Furcal vestige with four mesosetae and four apical microsetae in the apex of a small tubercle (Figs 9, 10). Female genital plate with 6 + 6 pregenital, 28 circumgenital and two eugenital setae (Fig. 11), genital plate of the only male studied with 6 + 6 pregenital, 16 circumgenital and 2 + 2 eugenital (Fig. 12), but it should be 6 + 6, 22, and 4 + 4 respectively. Each lateral anal valve with subcuticular reticulation, 11 setae and 2 microsetae. Ventro-internal tubercle of Abd. V well-developed and with strong subcuticular reticulation, one macroseta and three mesosetae.

##### Etymology.

The new species is named *H. centraliamericana* sp. n. for its distribution in Central America (Honduras and Costa Rica), but it might be even more widely distributed, as the two localities are approximately 800 km from each other.

##### Discussion.

This species has the unique characters of this new genus: the fusion of clypeal and antennofrontal tubercles and of dorsointernal and dorsoexternal tubercles on head. Additionally, the presence of only one tubercle on each side of the abdominal segment V is unique among Sensillanurini. The new species has more abundant head chaetotaxy than members of *Americanura* and *Palmanura*, including the antennofrontal, dorsolateral and lateral cephalic tubercles, and Th. I which has no Di seta, against one in all species of the genus *Sensillanura* (Palacios-Vargas and Catalán 2010). The presence of nine setae on Ant. I have been cited in other member of the Neanurinae (Deharveng 1981), here, there are five dorsal slightly barbulate macrosetae on a surface with subcuticular reticulation like in the *Neanura*, *Monobella* and *Neanurella* species which exhibit this character, and which belong to different evolutionary lineages; the 6 + 6 pregenital setae is also a character unique in the tribe. The furcal vestige of the new species is like that of *Sensillanura*, but more developed, as a small tubercle similar to that of *Morulina* species, with mesosetae and microsetae.

Variation: The ag setae in females varies from 5-6 pairs, and circumgenital ones from 15 to 28 setae. One teratologic specimen lacks left tubercle of abdominal segment VI. Some of the mesosetae on Di tubercle of Th. II and III are very thin and smooth and can be overlooked. The juvenile paratype has ten setae on anal valve instead of eleven. The specimens from Costa Rica have the dorsal macrosetae and mesosetae acuminate.

## Supplementary Material

XML Treatment for Honduranura

XML Treatment for Honduranura centraliamericana
